# siRNA and mRNA-Based Preventive and Therapeutic Strategies for HPV-Induced Cervical Cancer

**DOI:** 10.34172/apb.025.45456

**Published:** 2025-07-28

**Authors:** Mansi Khari, Neha Jain, Shreya Kaul, Manisha Pandey, Nitin Sharma

**Affiliations:** ^1^Department of Pharmaceutics, Amity Institute of Pharmacy, Amity University, Noida (U.P.), India; ^2^Department of Pharmaceutical Sciences, Central University of Haryana, Mahendergarh, India

**Keywords:** Gene silencing, Oncogenes, RNA interference, Targeted gene delivery, Gene knockdown

## Abstract

Human papillomavirus (HPV), specifically types 16 and 18, is the main cause of cervical cancer and a significant cause of death among women. Specifically, HPV E6 and E7 oncogenes hinder the normal cell cycle regulation, resulting in uncontrolled cell growth and cervical cancer. The available therapy options include surgery, radiotherapy, and chemotherapy, which show success but also demonstrate notable complications. SiRNA (small interfering RNA) and mRNA (messenger RNA) therapies have emerged as precise and effective tools to silence the HPV E6 and E7 oncogenes and stimulate the immune system to fight against HPV infection, respectively, presenting a targeted therapy approach and overcoming the available therapy challenges. Nanoparticles and Pegylated liposomes are the delivery systems that increase the efficacy and safety of siRNA and mRNA therapies. This review critically appreciates the effective targeting of siRNA and mRNA-based therapies by highlighting their key advantages and limitations. Despite being a target-specific and effective approach, there are certain challenges like scale-up, cost-effectiveness, and developing stable delivery systems, which are required to be discussed. In addition, other precision medicine approaches, such as CRISPR/CAS-9, antisense oligonucleotides, or immunotherapy, have also been included as compared to siRNA/mRNA therapies. Their preclinical, patent, and clinical translations have also been discussed exhaustively.

## Introduction

 Human papillomavirus (HPV), a naked, dual-stranded, circular DNA virus, is responsible for diverse epithelial lesions and malignancies. The likelihood of HPV infection is very high among sexually active individuals, as it is transmitted via sexual activities, penetrates the epithelial tissue, and attacks the basal stem cells. Without an HPV infection, cervical cancer doesn’t occur.^1^ HPV embeds in the cervix, the basal region of the uterus, resulting in the uncontrolled growth of cervical cells.^2^ There are about 100 subtypes of HPV, and the prevailing categories for HPV infection are mucosal or anogenital, non-genital (cutaneous), and epidermodysplasia verruciformis (EV). HPV infection can affect the skin, throat, and genital area, causing genital warts (small bumps).^3^ Cervical cancer can develop 12-15 years after the primary infection. Oncogenic HPV DNA is found in 95% of cervical cancer worldwide, which means HPV is responsible for 5.2% of all cancers. 75% of cervical carcinomas are attributed to HPV strains 16 and 18.^4-6^

 HPV infection rates are high in growing countries, and cervical carcinoma represents the foremost etiology of cancer-related mortality. Occupying the fourth position among the leading causes of cancerand death among women, HPV-induced cervical carcinoma is a global health crisis.^7^ According to the U.S. data, it is evident that sexually active women between the ages of 20-25 years, who have high HPV infection, have been found with the highest rate of HPV infection.^8^ In 2019, the World Health Organization (WHO) documented 620,000 new cancer cases in women. The incidence and mortality rates for cervical cancer in 2022 were 660,000 and 350,000, respectively.^6^ Between 2018 and 2030, it is expected that there will be 700,000 newly diagnosed cases of cervical carcinoma, up from 570,000.10 In 2020, 194 countries accepted the WHO’s global strategy to eradicate cervical carcinoma. Under the worldwide elimination strategy, WHO formed the Cervical Cancer Elimination Modelling Consortium (CCEMC) at the World Health Assembly in May 2020. The CCEMC models anticipated that 21.3 million cervical cancer cases would occur between 2020 to 2060. WHO urges all countries to ensure that vaccination, screening, and treatment continue with all precautions and safety. The role of vaccination for prevention and programs for screening is to detect early and treatment of HPV-induced cervical cancer.^8^

 HPV 16 and 18 cause 70% of cervical cancer. The main etiological factors comprise smoking, low immunity, early sexual activity onset, and several sexual partners, while the cofactors are tobacco smoking, prolonged use of contraceptives, and high parity^9-11^. The interaction with other STD infected persons leads to cervical cancer.^12^ The International Collaboration of Epidemiological Studies of Cervical Cancer (ICESCC) determined a woman’s number of sexual partners as an estimate for her risk of HPV exposure before examining the impact of hormonal contraceptives and other exogenous cofactors on cervical cancer risk. High parity (women who gave birth after the age of 35) maintains the area of the exocervix that is transformed for a prolonged time, which increases the exposure to HPV infection. People who smoke tobacco have a higher risk of getting cervical cancer than non-smokers, as it reduces the immune response in the cervical area, directly damages the genes, and leads to cervical cancer.^13^ Contraceptives are one of the cofactors, as the presence of estrogen and progesterone facilitates the HPV gene expression in the cervical area of the uterus through hormone release mechanisms in the viral genome.^14^ HIV-positive women exhibit increased susceptibility to HPV infection, as HIV reduces the immune response system of the body.^15,16^

 For the diagnosis of cervical cancer, firstly physical examination is carried out, which is different for a specific type of warts like cutaneous warts are examined at the underside of the toes anogenital warts are diagnosed by speculum check of anal/vaginal walls, while cervical dysplasia is checked by speculum in cervix Additionally, a preliminary or repeated Pap smear may be necessary, considering the patient›s age and Pap smear record.^17^ Patients having data about glandular warts or intraepithelial squamous warts may move forward with colposcopy, based on therapy assessment.^18^

 The treatment strategy depends upon the stage and extent of cervical cancer. Surgery, radiation therapy, chemotherapy, and immunotherapy are the treatments used in HPV cervical cancer. Other treatment options that are currently used include sentinel lymph node biopsy, pelvic lymphadenectomy, and chemoradiotherapy.^19^ Surgeries are mostly used as successful techniques in the early stages of cancer are being adopted by doctors. Loop electrosurgical excision procedure (LEEP), radical hysterectomy, total hysterectomy, cryosurgery, and trachelectomy are the spectrum of surgical modalities employed in the current management of cervical cancer.^20^ Nevertheless, there are serious side effects associated with the abovementioned therapies that may affect patient’s short- and long-term health. Chemotherapy and radiation therapy are non-specific, thus impacting healthy cells as well. There is a high probability of drug resistance with chemotherapy and cancer recurrence with radiation therapy, owing to the presence of microscopic cancer cells. In addition, a hysterectomy may cause long-term bleeding and hormonal imbalance, which may have an impact on the emotional and psychological health of an individual. Consequently, there is an increasing interest in less intrusive and more focused methods, like immunotherapy, siRNA (small interfering RNA), and mRNA (messenger RNA)-based therapies, which may provide more precise and less harmful choices for the management of cervical cancer.^21^

 Considering their accuracy and ability to target cancer-specific pathways, siRNA and mRNA therapeutics have attracted significant consideration in cervical cancer therapy.^22^ The siRNA technique is used to target and degrade HPV mRNA present inside the infected cells,^23^ as siRNA sequences suppress the HPV 16, E6, and E7 oncogenes, which cause cervical cancer.^24^

 On the other hand, mRNA therapy directs the immune system to attack and kill cancer cells. mRNA-based vaccinations work by directing cells to synthesize antibodies. The translation of the mRNA into proteins (such as the oncogenic proteins of HPV E6/E7 or other antigens specific to cancer) occurs once the mRNA enters the cells. When these proteins are seen on the surface of cells, the immune system is prompted to recognize and eradicate cancer cells that are either HPV-positive or malignant.^25^ They efficiently attenuate cancer cell’s viability via targeted RNA interference. These techniques overall inhibit the proliferation of HPV 16-positive carcinoma cells.^26^ This review critically evaluates siRNA and mRNA therapies for HPV-induced cervical cancer, addressing their efficacy and limitations relative to conventional treatments.

## Selection of literature

 PubMed, Springer, Google Scholar, and Science Direct were searched to find research articles from the last five years, i.e., 2020-2025. Two reviewers independently screened the title and abstract with consideration of keywords such as “siRNA”, “mRNA”, “siRNA drug delivery in cervical cancer”, mRNA drug delivery in cervical cancer”, “cervical cancer therapy”, “si-RNA formulations”, mRNA-based formulations” and more with a combination of “OR” and “AND”. Cross-referenced articles were another tool used to gather further information, and discrepancies were eventually resolved through discussion. The selection criteria were the research papers having *in vitro* cell line studies, and/or preclinical studies, while review papers, editorials, conference proceedings, and other language articles were excluded.

## Pathophysiology of HPV-induced cervical cancer

**Figure 1:**
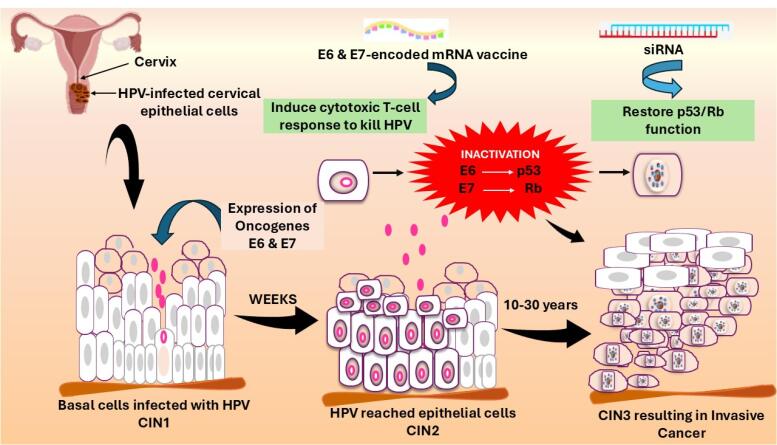
Schematic illustrating HPV pathogenesis, RNA, and siRNA therapy mechanisms

 HPV disrupts the mucosal surface and infects the cervix epithelial cells. Figure 1 highlights the whole pathogenesis of HPV-associated cervical carcinoma.^27^ The disruption of the normal cell cycle promotes irregular cellular proliferation. In the cervix, the invasive squamous cell carcinoma leads to the disease’s pre-invasive stage, in which the abnormal cells are restricted to the epithelium. The disease’s non-invasive stage is called cervical intraepithelial neoplasia (CIN).^28^ HPV has two special phase genes, i.e., 7 early (E) and 2 late (L) phases in its genomic sequence, which cause necessary viral proliferation.^29^ The viral DNA undergoes genomic integration in the host cell, which causes the disruption of viral oncogenes E6 and E7. These proteins disable tumour suppressor proteins p53 and retinoblastoma (Rb), causing genetic mutation.^30,31^ This mutation, with time, leads to neoplastic transformation of the epithelium. The premalignant lesions progressed to cervical carcinoma. This is the initial stage of the cancer, i.e., CIN1. It is a mild dysplasia having limited abnormalities that affects the lower region of the epithelium and resolves within 9-12 months. CIN1 shows a variable risk of growing into CIN2 and CIN3. CIN2 is a type of moderate dysplasia that extends into the upper portion of the lower layer of the cervix. It is treated by excision or ablation due to its progression. CIN3 is a severe dysplasia that involves the whole thickness of the epithelium, leading to the development of cancerous cells in that part. The risk of cancer progression is higher for CIN3 than for CIN1 and CIN2. The micro-invasive carcinoma is present in a CIN3 background defines its malignant potential.^32^ The tissue structure of the wart may show signs of parakeratosis, i.e., keratinization of cells in the ectocervix, papillomatosis, i.e., growth of lumps, and hyperkeratosis.^33^ i.e., the appearance of white patches on the cervix.^34^ The capillaries are frequently thrombosed, and the long rete lines usually indicate the wart origin.^35^

###  Symptoms

 The most common symptom that can be seen in women is abnormal vaginal bleeding between periods or after menopause. Pelvic pain also occurs in advanced stages and affects the nearby tissues.^36,37^ Dyspareunia (pain during intercourse) increases due to physical changes in the cervix. A lump can be seen or felt in the pelvic area. Vaginal discharge, which can be watery, mucoid, and malodorous, can be observed.^38^ These symptoms can also be caused by other diseases. Evaluation and appropriate testing with screening should be done for HPV cervical cancer.^39^

 Immunohistochemistry, which involves p16INK4a, is a biomarker used for HPV related cervical lesions by reflecting E7-E7-mediated Rb inactivation. HPV DNA testing is also used to diagnose and manage lesions, leading to treatment. There are different types of antigens present in cervical carcinoma, which are detected by using immunohistochemical techniques on tissue sections.^40^ A tabulated presentation of all cervical cancer biomarkers and their detection techniques is mentioned in Table 1.

**Table 1:** Compilation of biomarkers involved in cervical cancer and their detection methods^40^

**Biomarkers**	**Detection method**
CEA (Carcinoembryonic antigen)	Radioimmunoassay, ELISA
CA 19-9	Radioimmunoassay, RIA
CA125	Transvaginal ultrasonography
SCC antigen (Squamous cell carcinoma antigen)	Serum tumour marker assay
CYFRA (cytokeratin 19 fragment)	Enzyme-linked immunosorbent assay (ELISA)
*nm23-H1*	Western blotting, ELISA
c-erbB-2 (HER2/neu)	Fluorescence in situ hybridisation (FISH), quantitative Polymerase chain reaction
HIF-1ɑ (Hypoxia-Inducible Factor 1 alpha)	Quantitative reverse transcription PCR (qRT-PCR)
VEGF (Vascular Endothelial Growth Factor)	Flow cytometry
CA9 (Carbonic Anhydrase IX)	Mass spectrometry
COX-2 (Cyclooxygenase-2)	Quantitative reverse transcription PCR
c-myc (oncogene)	Immunocytochemistry
ras (oncogene)	GTPase activity assays
Estrogen and progesterone receptor assays	Radio-labelled ligand binding
DNA ploidy and S-phase	Flow cytometric analysis

## Cervical cancer: Current treatment modalities

 The management and treatment plan for cervical carcinoma is determined by its progression and spread. Table 2 highlights the available therapy options along with their description. The current treatment’s main goal is to curtail the chance of developing invasive cervical cancer in the future, remove the wart transformation zone, and improve symptoms. The management and treatment depend on the HPV types and progression of the disease. Radiation therapy, a combination of surgery and chemotherapy, is the strategy against HPV cervical cancer.^41,42^

**Table 2:** Treatment strategies employed for the management of HPV-induced cervical cancer^43^

**S.No.**	**Treatment**	**Description**
1.	Total hysterectomy	Surgical procedure for the entire uterus with the cervix removal under anesthesia. Oophorectomy: the removal of ovaries called as is also done in women who have completed childbearing. Total hysterectomy can be performed through different approaches depending on the condition of the cancer growth.
	Abdominal hysterectomy	The incision in the lower abdomen for the removal of the uterus.
	Vaginal hysterectomy	The removal of the uterus through the incision in the vagina.
	Laparoscopic or robotic hysterectomy	A small incision in the abdomen for the removal of the uterus using specialized instruments and a camera.
2.	Radical hysterectomy	An extensive surgical procedure in which the entire uterus is removed with parametria, cervix, and upper part of the vagina. It is used for larger cervical lesion sizes of more than 2 cm.
3.	Loop Electrosurgical Excision Procedure (LEEP)	A thin wire loop heated by an electrical current cut out the cervical tissue demonstrating dysplastic features. In an anesthetized state, to numb the cervix. The benefit of sealing the blood vessels as it cuts leads to a decrease in the bleeding.
4.	Conization	Surgical resection of a cone-shaped specimen from cervical tissue.
5.	Trachelectomy	Surgical procedure for removal of the cervix but preserving the uterus to maintain fertility.
6.	Cryosurgery	Involves a liquid nitrogen-cooled metal instrument applied on the cervix to freeze and induce apoptosis by creating ice crystals.
7.	Laser therapy	Focused laser beam to destroy the abnormal tissue or high-grade lesions.
**Types of radiation therapies**		
8.	External beam radiation therapy (EBRT)	High-energy radiation is targeted to destroy the tumour cells in the cervix while preventing the surrounding cells.
9.	Intensity-modulated radiotherapy (IMRT)	A combination of photon and proton radiation beams to ablate cancerous cells according to their shape.
10.	Brachytherapy (internal RT)	A radioactive source is implanted inside or at the site of the tumour, then irradiated with high-dose radiations, sparing the surrounding tissue.
**Chemotherapy-based treatment**		
11.	Cisplatin with 20% topotecan	Combination of cisplatin and topotecan by damaging the DNA of cancer cells and inhibiting the topoisomerase, preventing the repairing of damaged DNA respectively. Thus, a synergistic effect can be obtained which reduces the topotecan dose i.e. 20% and its associated side effects.
12.	Cisplatin combined with paclitaxel	Combination therapy of cisplatin and paclitaxel damages the cancer cells and acts as a mitotic inhibitor, respectively. This causes inhibition of cancer cells from proliferating
13.	Chemoradiotherapy	Combination of chemotherapy and radiation: targets rapidly dividing cancer cells by inhibiting replication and damaging the cancer cell DNA and high-energy radiation targets specific site of tumour cells sparing the surrounding cells.
14.	Palliative chemotherapy	Drugs are used to provide symptomatic relief and enhance well-being.

 Chemotherapy is the standard course of treatment and is used as additional therapy after surgery when the risk of recurrent disease increases due to poor features of the tumor. It is also administered in combination with radiotherapy. For the last three decades, the single agent demonstrating maximal effectiveness when utilized as monotherapy is the platinum-based chemotherapeutic, cisplatin.^44^ The resistance is developed while undergoing therapy, which reduces the efficacy of platinum-derived chemotherapeutics.^45^ Studies show that cisplatin demonstrates greater synergistic effects in combination than as monotherapy.^46^

 Specifically, radiotherapy and chemotherapy together are associated with enhanced treatment-related adverse effects, as revealed in a study on 1,030 subjects. Fifty-eight percent of patients revealed gastric complications, which include nausea, vomiting, and radiation-induced enteritis. A total of 78.5% of patients experienced neurological symptoms, with the most common being approximately 35% pain, and twenty percent asthenia.^47^ Furthermore, owing to drug resistance that occurs during tissue infiltration and metastasis, around ninety percent of cervical cancers with resistance to chemotherapy do not respond to existing therapies. After receiving radiation treatment, the likelihood of recurrence for advanced-stage patients might range from fifty percent to seventy percent after two years.^48^

 The nature of complexity, surgical technique, and patient vulnerability all affect the incidence of hysterectomy consequences following chemotherapy for cervical cancer. As observed in a randomized clinical trial on stage IB cervical cancer-suffering women, dual treatment with radiation along with chemotherapeutics, two percent of patients experienced ureteral stricture, while it became five percent for stage IIA patients with symptoms like rectal and small-bowel stricture.^43^ In another study, hysterectomy of different sites results in different infection rates, i.e., vaginal shows four to ten percent, while abdominal shows six to twenty-five percent.^49^ Post vaginal hysterectomy, up to thirteen percent of patients experience infectious problems, but only nine percent do so following minimally invasive surgery.^50^

## HPV vaccination: The first line of defence

 Lifestyle changes can dramatically decrease the risk of acquiring cervical cancer. Vaccination is a preventive strategy against the HPV virus to treat cervical cancer, when paired with the appropriate use of contraceptives and a sexually healthy lifestyle. Precancerous lesions are due to HPV serotypes 16 and 18, both of which are the focus of most vaccine development, to limit the occurrence of cervical cancer in both men and women. The vaccines used E6 and E7 as the target antigens, which work by producing cell-mediated immunity instead of neutralizing antibodies, leading to immediate therapeutic effect in already affected patients.^51^ Several vaccines targeting E6 and E7 are being developed or are under clinical trials, including live nucleic acid-based vaccines, peptide-based vaccines, and vector vaccines. Virus-like proteins (VLP)-based vaccines have been under clinical trials for over past five years. The trials are for the specific type of protection from the growth of cervical dysplasia. Adolescent or pre-adolescent girls could benefit from this HPV-VPL prophylactic vaccination program if it is given before the start of their sexual life.^51,52^

 The accomplishment of these vaccination programs is challenging at the current time due to the lack of public health interventions that can ensure regular clinic attendance among pre-adolescent girls. The HPV vaccine may have different efficacies in the two genders, as there are chances for the vaccine to have less inhibition of the growth of infection in males than in females.^52^

 People stigmatize HPV infection as other sexually transmitted diseases, due to which parents do not consider vaccination for girls during adolescence. The problem associated is the general public›s unawareness of HPV and its relation to cervical cancer, or the requirement of getting vaccinated.^53^ They lack the knowledge necessary for the vaccination against HPV.

 Currently, there are three available vaccines against HPV produced by a recombinant DNA technology containing virus-like particles (VLPs) as given in Table 3.^54^ They cause the immune system to react strongly, and natural infections result in increased antibody titres. Gardasil R is the first commercially available vaccine, manufactured under Merck & Co., Kenilworth, NJ, USA, and approved by the FDA in 2006. The four HPV serotypes, i.e., 6, 11, 16, and 18, are targeted by the quadrivalent vaccine. The vaccine is for both females and males under the age of 9-26 years. Cervarix is a bivalent HPV vaccine manufactured under GlaxoSmithKline that received approval by the EMA, i.e., European Medicines Agency, in 2007 and by the FDA in 2009. It protects from two HPV serotypes, 16 and 18, which are the main cause of cervical cancer. Cervarix is for both females and males in the age group of 10-25. A non-avalent vaccine called Gardasil 9 is a new vaccine approved by Merck EMA. It covers the four original types of serotypes of Gardasil R and five more oncogenic serotypes, HPV 31, 33, 45, 52, and 58. It can protect from most cervical cancers. It leads to a longer interval of time between the screenings for cervical cancer, which saves a lot of money. More than 60% of countries have HPV coverage, such as Australia, Denmark, and Sweden. Over 1% of teenage girls in low-income countries get HPV vaccinations, but the remaining are still unprotected. To overcome this, the implementation in these countries of HPV vaccines is urgently required.^55^

**Table 3:** Different types of vaccines and their characteristics available for cervical cancer prevention

**Vaccine types **	**Vaccine **	**Route of Administration **	**Schedule of injection **	**HPV strains covered **	**Response **
Quadrivalent vaccine	Gardasil R	Intramuscular injection	Three doses at 0, 2, and 6 months	Targets four serotypes 6, 11, 16, and 18	Protection against genital warts, vulva, and cervical precancerous lesions
Bivalent vaccine	Cervarix R	Intramuscular injection	Three doses at 0, 1 and 6 months	Targets two serotypes HPV 16 and 18	Prevents the precancerous lesions and produces antibody titres
Non-avalent	Gardasil 9	Intramuscular injection	Three doses at 0, 2 and 6 months	Targets serotypes HPV 6, 11, 16, 18, 31, 33, 45, 52 and 58	Shows higher efficacy than Gardasil as it provides broader protection

###  Actionable Solutions against cervical cancer vaccine access challenges

 As discussed above, low- and middle-income countries have limited access to these costly cervical cancer vaccines, but the coupling of innovative approaches, cost-reduction strategies, along with evidence-based solutions can help to overcome these issues. In the United States, vaccines for children and endorsing low- and middle-income country funding via campaigns like Gavi can help to minimize the financial burden.^56^ Gavi is a worldwide foundation that enhances the use of the latest and limited-use vaccines specifically for children residing in the poorest countries of the globe. In addition, using altruistic pricing and indigenous manufacturing, India›s Serum Institute created Cervavac, a quadrivalent HPV vaccination that costs $2.50 to $5 per dose.^57^ Furthermore, the innovations in vaccination approaches can help to overcome the above-mentioned challenge. There are around 20 therapeutic vaccines in the pipeline of the World Health Organization that can be used in the therapy of existing HPV infections, thus benefiting the already infected women and supporting the preventive vaccine.^58^ scientists are exploring vaccines beyond E6/E7 oncoproteins, i.e., HPV E1 and E2 antigens. VTP-200, a multiantigen therapeutic vaccine, is in Phase 1b/2 trials. This vaccine targets five types of HPV, specifically the conserved domains of E1, E2, E4, E5, E6, and E7.^59^ The introduction of mobile-health units and school-based vaccination programs can enhance the acceptance of vaccination. In Ohio, mobile clinics had delivered HPV vaccines under the flagship program, i.e., the 513 Relief bus, and directly aided in self-collection HPV testing to low-resource settings by removing mobility constraints. The same approaches can be applied in low- and middle-income countries.^60^ In Australia and Rwanda, the implementation of HPV vaccinations at the school level, coupled with knowledge and training, helped to delay adolescents’ prior onset of their sexual activity.^61^

## SiRNA and mRNA-based cervical cancer therapies

 Molecular biology has seen some significant developments in recent years. Regulation of gene expression by short non-coding RNAs represents a single component within the multifaceted nature of this transition.^62^ Small non-coding RNAs are divided into three primary groups according to their physiological roles and arrangements: miRNAs, siRNAs, and piRNAs. siRNA or small-interfering RNA is a class of double-stranded, non-coding RNA with an approximate length of 20–23 nucleotide base pairs. It works by preventing the gene with the corresponding sequence from being expressed.^23^ Progress in comprehending genetic activities in plants and animals is attributable to the potent gene-silencing properties of siRNA. The therapeutic potential of siRNA in cervical cancer treatments stems from its ability to selectively bind and degrade complementary mRNA sequences, resulting in the inhibition of oncoprotein synthesis.^62^ In cervical cancer treatment, siRNA silences E6 and E7 oncogenes, which further restores p53 tumor suppression protein and pRb protein, thus leading to arrest of the cell cycle, cell death, and proliferation of tumor cells.^63^

 This mechanism of siRNA makes it suitable for cervical cancer therapy, like silencing of specific oncogenes or the genes responsible for drug resistance, and is highly specific to mRNA transcripts of disease-causing genes. Being highly specific, the risk of systemic toxicities or adverse effects is reduced significantly.64 siRNA-mediated delivery has shown no or low off-target impacts in HPV-negative cells.^24^ On the other hand, there are certain challenges as well while using these siRNA-based therapies. Nucleases and phosphatases are the major enzymes that cause the quick degradation of siRNA during its systemic circulation, which diminishes therapeutic activity. In addition, siRNA undergoes endocytosis, which results in degradation in endosomal compartments. Furthermore, it is difficult to scale up the production and delivery of siRNA, confirming uniformity in therapeutic effect.^65-67^ A single-stranded molecule, i.e., mRNA (messenger RNA), transports the genetic information from the nucleus DNA to ribosomes, so that proteins can be formed. mRNA-based vaccines are encoded with HPV antigens that trigger the immune system to induce a cytotoxic T-cell response. The best part of mRNA is that its vaccine development is rapid and cost-effective, owing to an established, scalable production process and *in vitro* transcription.^68^ In addition, these vaccines don’t carry any killed or live antigens, thereby minimizing the risk of severe adverse effects.^69^ Furthermore, other immunotherapies like immune checkpoint inhibitors can be combined with mRNA vaccines to improve the therapy outcome.^25^ However, being a cost-effective and easily scalable option for cervical cancer therapy, it is pertinent to mention that tumours show heterogeneity based on genetic information, resulting in the complicated selection of identification of broadly beneficial antigens for mRNA vaccines.70 In addition, cervical cancer escapes from immune system identification by diminishing antigen presentation, hence impairing vaccination efficacy.^71,72^ Preliminary clinical trials revealed variable outcomes, i.e., in the mRNA vaccine trial for pancreatic cancer, approximately half of the subjects had an immune response, with effectiveness correlated with spleen retention.^73^ Likewise, animal studies show promising results in cervical cancer, but during its translation may activate the immune system.^71^

## Comparison between siRNA and mRNA-based therapies with other precision medicines

 In recent years, there has been significant progress in the development of advanced molecular and immunotherapeutic approaches for the prevention and treatment of HPV-induced cervical cancer. SiRNA and mRNA-based platforms have emerged as promising strategies due to their ability to precisely target HPV oncogenes and stimulate a strong immune response.^74^ However, other novel modalities such as clustered regularly interspaced short palindromic repeats (CRISPR)/Cas9, antisense oligonucleotides (ASOs), immune checkpoint (IC) inhibitors, chimeric antigen receptors (CAR)-T Cell Therapy, oncolytic virus therapy, and epigenetic therapies are also being explored for their potential to overcome the limitations of conventional treatment.^75^ A summary Table 4is included to highlight key studies supporting each modality. Scalability, cost, and regulatory challenges are important factors that affect how practical it is to use siRNA and mRNA-based therapies for treating HPV-induced cervical cancer, especially in low- and middle-income nations (LMICs), where the disease is most common.^75,76^

**Table 4:** Comparison between siRNA, mRNA, and other precision therapy approaches

**Technology**	**Description**	**Mechanism of action against cervical cancer**	**Research study**	**Outcomes**	**Limitations**	**References**
CRISPR/Cas9	A gene-editing tool that uses guide RNA and Cas9 to create targeted DNA double-strand breaks for precise modification.	Targets and disrupts HPV oncogenes (E6/E7), restoring tumor suppressor functions (p53 and Rb), leading to apoptosis or senescence of cancer cells.	To develop CRISPR/Cas9-based pH-responsive nanovectors for HPV gene editing for cervical cancer and enhance T-cell therapy.	Constructed pH-responsive NPs for delivering Cas9 mRNA and guide RNAs targeting HPV E6/E7 oncogenes. Demonstrated effective gene editing, tumor suppression, and improved CD8 + T cell survival with adoptive T-cell transfer.	Limited *in vivo *sequencing confirmation, scalability challenges, and need for further optimization of combination therapies and long-term safety.	^77-79^
			To evaluate CRISPR/Cas913a targeting HPV18 E6 mRNA for cervical cancer therapy and synergy with cisplatin.	Effective E6 knockdown, p53 restoration, increased apoptosis, and enhanced cisplatin efficiency.	No *in vivo *validation, limited evaluation of antiviral and anticancer effects on other HPV genes (e.g., E1, E2, L1, L2).	
Antisense Oligonucleotides (ASO)	Short, synthetic single-stranged DNA or RNA molecules designed to bind specific mPNA sequences, modulating gene expression.	Binds to HPV E6/E7 mRNA, promoting RNase H-mediated degradation or blocking translation, thereby inhibiting oncogene expression.	To investigate role of allyl isothiocyanate in enhancing miR16 in HeLa-derived exosomes and its impact on fibroblast, apoptosis, angiogenesis and inflammation within TME.	Increased miR16 expression, upregulated apoptotic markers (Bax/Bcl2 ratio), and downregulated angiogenic and inflammatory markers.	Limited to *in vitro *models, and the detailed molecular mechanism was not fully elucidated. Patient-derived samples were not assessed for miR16 modulation.	^80-82^
			Develop AmNA-modified ASO (8-3419) targeting MCM8 as a cancer-specific chemosensitizer for platinum compounds.	Sensitized cancer cells to cisplatin and Olaparib by inhibiting MCM8 *in vitro *and *in vivo. *Supressed tumor growth in HeLa (cervical cancer) and HCT116 (colon cancer).	Moderate growth inhibition of cancer cells. Off-target effects in earlier versions (AS) 8-3); delivery improvements needed for better *in vivo *efficacy.	
Immune checkpoints (IC) inhibitors	Monoclonal antibodies targeting immune checkpoint molecules (eg., PD-1, CTLA-4) block inhibitory pathways in T-cells, enhancing immune response against tumors.	Inhibits PD-1/PD-L1, CTLA-4 pathways, restoring T-cell activity to recognize and destroy HPV-transformed cervical cancer cells.	To assess how doxorubicin and docetaxel affects the affects IC genes PD-L1, VISTA and CTLA-4 in cervical carcinoma cell lines and understand how these checkpoints are regulated, so as to optimize immunotherapy strategies.	Doxorubicin upregulated PD-L1, CTLA-4 and downregulated VISTA, highlighting its impact on IC and supporting combination immunotherapy.	Limited to single cell line. Does not explore downstream functional impacts of gene expression changes or confirm protein-level effects.	^83-85^
			To evaluate CTLA-4 and CD137 IC molecules in cervical tissues as targets for immunotherapy in HPV-associated cervical carcinoma.	CTLA-4 was positive in 32% of cancers, whereas CD137 was positive in 91% of immune infiltrates. CTLA-4 was significantly higher in cancer vs benign tissue.	Lack of functional assays. Study limited to expression analysis without testing therapeutic efficacy or biological function. No clinical outcome data.	
Chimeric antigen receptors (CAR)-T cell therapy	Genetically engineered T-cells expressing CARs that recognize specific antigens on cancer cells.	Engineering T-cells with CARs that target specific tumor antigents, such as HPV E6/E7 proteins, leading to T cell activation, cytokine release and tumor cell lysis through apoptosis.	To develop CAR-T cells using TCR mimic nanobody targeting HPV16 E6 presented by human leukocyte antigen (HLA)-A∗02:01.	Demonstrated specific killing of HPV16 + cervical cancer cells *in vitro *and tumor reduction in mouse models. They showed high specificity without affecting HPV-negative or (HLA)-A∗02:01-negative cells.	Tested only in immunodeficient mice. Applicability limited to patients expressing HLA-A∗02:01and infected with HPV16, restricting broader clinical utility.	^86-88^
			Develop and assess placental alkaline phosphatase (PLAP) targeted CAR-T cells for HPV-induced cervical carcinoma.	Demonstrated antigen-specific activation, cytokine secretion and cytotoxicity against PLAP + ve cervical cancer cell lines. Target-specific killing of HeLa and CaSki cells.	Potential on-target off-tumor toxicity due to low-level PLAP expression in some normal tissues was not fully addressed.	
Oncolytic virus therapy	Utilizes genetically modified virus that selectively infect and kill cancer cells while stimulating anti-tumor immunity.	HPV replicates in cervical cancer cells, causing cell lysis and releasing antigens and viral particles that stimulates the immune system. This response targets the virus and enhance the destruction of uninfected cancer cells.	Develop and evaluate the efficacy of a recombinant oncolytic herpes simplex virus (HSV) type 1 using a CRISPR/Cas9 system targeting HPV16 oncogenes.	Reduced E6/E7 expression, restored P53/pRB, inhibited tumor growth, induced apoptosis and caused G2/M cell cycle arrest.	Complete eradication of all integrated HPV16 copies as not achieved. Potential for incomplete or off-target editing not fully ruled out.	^89-91^
			Evaluated a combined therapy using oncolytic HSV with BEZ235 and epacadostat for enhanced immune response against HPV-induced cervical cancer.	Reduced tumor size, increased CD8^+^T cell infiltration and strong synergistic immune activation.	Conducted in immunocompetent mouse models, which may not fully mimic human cervical cancer complexity. Long-term safety and resistance not fully addressed.	
Epigenetic therapies	These modify gene expression by targeting DNA methylation and histone modifications, reactivating tumor suppressor genes and inhibiting cancer cell growth while enhancing therapeutic response,	Reverse hypermethylation or histone modification in HPV-infected cells, restoring tumor suppressor gene expression and inhibiting oncogenic pathways.	To investigate an epigenetic therapy using targeted demethylation of the EphA7 promoter can restore its expression and supress cervical carcinoma progression.	The epigenetic reactivation of EphA7 via dCas9-TET1 reduced tumor growth and enhanced sensitivity to chemo-, radio- and immunotherapy by modulating SP1/DNMT1 and PI3K/AKT signalling.	Primarily preclinical; clinical safety, delivery and off-target effects of epigenetic editing remain untested in humans.	^92-94^
			To investigate the potential of Alpha-linolenic acid (ALA) in regulating cervical cancer through epigenetic mechanisms in HPV + ve and -ve cell lines.	Reduced expression of DNMTs, HDAC8, and histone methyltransferases. Increased expression of DNA demethylase, histone acetyltransferase, and methyltransferases. Downregulated oncogene hTERT expression.	Unclear mechanism for hTERT remains unclear. The exact molecular interactions of ALA with epigenetic regulators need further elucidation.	

 mRNA technologies can be produced on a large scale because they are made using synthetic, cell-free methods that do not require growing cells. siRNA is also made through chemical processes and is easier to produce in large amounts, but it has issues with delivery into the body, which makes widespread use more difficult.^95^ While the cost of production of RNA and delivery systems has decreased, both mRNA and siRNA treatments are still more expensive than traditional vaccines or small-molecule drugs.^96-98^ Lipid nanoparticles, commonly used to deliver RNA, also require cold storage, which is difficult to manage in many low-resource settings.99 Other newer therapies, including CRISPR/Cas9 and ASOs, provide targeted gene regulation but still face challenges related to safety, delivery, and long-term effects.^100,101^ IC inhibitors and CAR-T cell therapies are even more complex and costly, often requiring personalized treatment and advanced hospital facilities, making them mostly suitable for high-income countries.^102,103^ Oncolytic virus treatment and epigenetic therapy are promising in early studies, but their large-scale production and delivery remain under development.^104^

 Getting regulatory approvals for RNA-based cancer therapies is also a slow process, with guidelines to ensure safety, control immune response, and ensure stability. While the success of mRNA COVID-19 vaccines helped improve regulatory understanding of RNA technologies, using them for cancer treatment still faces challenges.^105^ In LMICs, these challenges are greater due to limited local production, weak regulatory systems, and trained professionals. To make siRNA and mRNA therapies more accessible in these settings, there is a strong need for investment in local manufacturing, improvement in cold-chain and delivery infrastructure, and global collaboration to reduce regulatory and financial barriers.^106^

## Recent advancements in SiRNA and mRNA-based cervical cancer therapies

**Figure 2:**
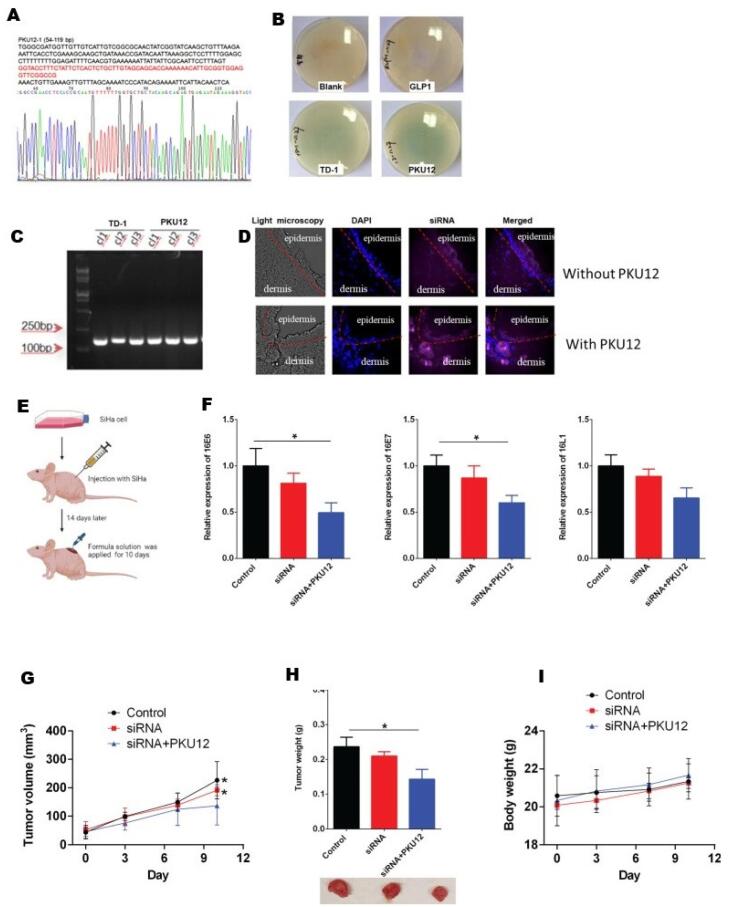
PKU12 (A) Sequence; (B) Transdermal capability assessment via blue, white screening; (C) Colony’s PCR assessment; (D) Fluorescent staining of the siRNA administered transdermally; (E) Outline of siRNA preclinical assessment; (F) qRT-PCR analysis of PKU12 siRNA and siRNA delivery on 16E6, 16E7, and 16L1 expression; PKU12-siRNA impact on (G) tumor volume (H) tumor weight (I) body weight.^107^

 A research study by Yan and group designed a novel transdermal peptide known as PKU12 based on the existing transdermal peptide TD-1, intending to investigate its potential in siRNA-based anti-tumor therapy for HPV, as shown in Figure 2. The research involved analysing the efficiency of PKU12 as a carrier of siRNA against HPV16 E6, E7 L1 and the effect on tumourigenesis in tetrathyl alcohol-induced oral carcinogenesis using nude mice. This research provided the first evidence that the peptide PKU12 could effectively facilitate the transdermal delivery of siRNA that targets HPV16 genes specifically, resulting in a marked reduction in mRNA levels of HPV16 E6, E7, and L1 in SiHa cell-derived xenografts. The treatment also significantly inhibited tumor growth. 586 differentially expressed genes (DEGs) were found in tumours as per the RNA-sequencing data, which were then treated with PKU12 and siRNA compared to control tumors. Gene set enrichment analysis (GSEA) indicated that these DEGs were negatively associated with pathways including HIF-1, TNF, AGE-RAGE, ferroptosis, NF-kappa B, IL-17, rheumatoid arthritis, and ovarian steroidogenesis. Subsequently, functional enrichment analysis on the DEGs suggested that these genes were enriched mainly in the IL-17 signaling pathways, TNF signaling pathways, and cytokine–cytokine receptor interaction pathways. As per the survival analysis, genes such as ZBTB16, MYH4, MYH1, DEPP1, and FGG were correlated with reduced survival duration in cervical cancer patients, while high expression of RAB3C, SULT1E1, PROX2, and CXCR3 was linked to better survival outcomes. Altogether, this work indicates that PKU12 can potentially be used as an siRNA transdermal delivery platform to provide a novel cervical cancer treatment.^107^

**Figure 3:**
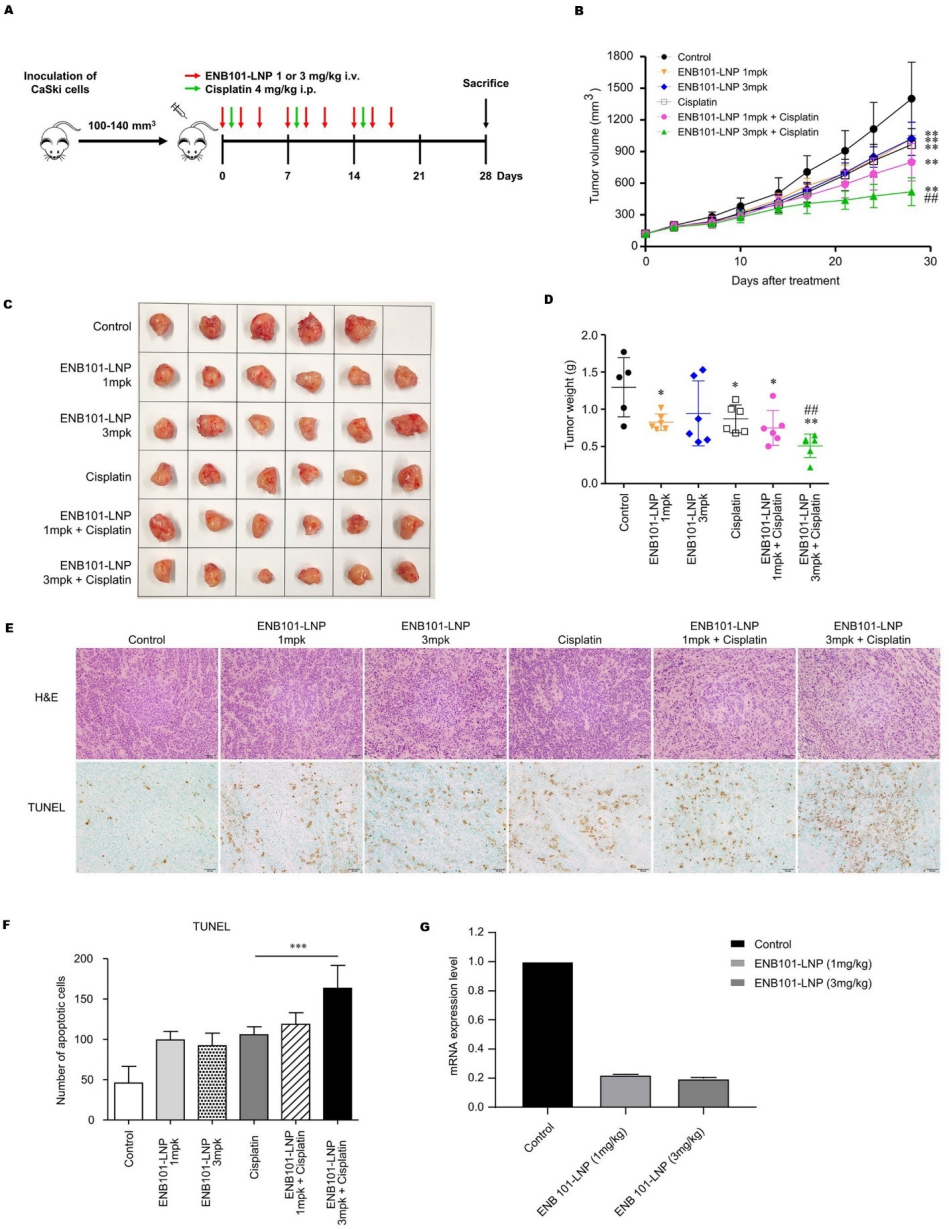
CaSki xenograft mouse model: Synergistic effect of cisplatin and ENB101-LNP. CaSki cells were administered into six weeks of age female nude BALB/c mice. (A) On Day 0, two weeks after CaSki cell incubation, when tumor sizes became 100–140 mm³, mice were segregated into a group of six. Three intravenous doses of ENB101-LNP were given for three weeks, while cisplatin was given via intraperitoneal injection weekly for the same period. (B) The rate of inhibition was calculated by generating the inhibition rate, with tumor volumes reported as mean ± standard deviation (Wilcoxon test, **P< 0.01 versus control, ##*P*< 0.01 versus cisplatin). (C) Tumors were harvested from mice implanted with CaSki cell xenograft. (D) Tumor specimens were weighed, and a statistical comparison of tumor weights was performed utilizing an unpaired t-test, revealing statistically significant differences (*P < 0.05, **P < 0.01, ##*P* < 0.01). (E) Histopathological analysis and TUNEL staining for apoptosis in tumors (magnification × 200). (F) TUNEL assay results showed significant apoptosis (unpaired t-test, ***P < 0.001). (G) Tumor samples from CaSki xenografts were collected post-6 treatments on day 11, and qRT-PCR was utilized to measure HPV16 E7 expression levels.^110^

 Selenium (Se) nanoparticles (NPs) appeared as promising vehicles for cancer drug and gene delivery due to their ability to prevent cancer, reduce drug toxicity, and support immune and thyroid function. SeNPs outperform other biogenetic and synthetic strategies, but their lack of active targeting remains a challenge. To address this, Yu and group developed the peptide RGDfC, which targets αvβ3 integrin overexpressed in cancer cells such as HeLa, and was attached to SeNPs, creating RGDfC-SeNPs. Following this, Derlin1-siRNA was incorporated into the RGDfC-SeNPs, resulting in the generation of RGDfC-Se@siRNA. These NPs› positive charge facilitates effective binding with siRNA via electrostatic interaction, enhancing targeted gene delivery. The developed NPs effectively targeted HeLa cervical cancer cells, inducing gene silencing and subsequent inhibition of cell migration, invasion, and proliferation. The NPs triggered apoptosis *in vitro* through mitochondrial dysfunction and increased ROS. Furthermore, *in vivo* studies utilizing murine xenotransplantation models utilizing HeLa cells confirmed the antitumor efficacy of these NPs while demonstrating negligible toxicity to major organs.^108^ RNA interference (RNAi) represents a significant therapeutic potential that targets oncogenes. In cervical cancer, RNAi has been utilized for the suppression of HPV›s E6 and E7 oncoproteins, which are indispensable for cancer progression. By inhibiting these proteins, RNAi can restore tumor suppressors like Rb and p53, leading to cell senescence or apoptosis. However, delivering siRNA effectively remains challenging owing to instability and limited cellular internalization. Lipid nanoparticles (LNPs) have emerged as a promising solution, enhancing siRNA stability and delivery to tumor sites.^109^ Therefore, the anti-cancer ability of ENB101-LNP, LNPs encapsulating siRNA-mediated knockdown of HPV16 E6/E7, was combined with cisplatin in cervical carcinoma models by Sung and colleagues (Figure 3). The results demonstrated that cisplatin and ENB101-LNP synergistically inhibited CaSki cell growth, reducing HPV16 E6 or E7 mRNA levels and increasing p53, p21 mRNA, p21, and HLA class 1 protein expression. In the mouse model, the combination treatment markedly suppressed tumor growth and induced apoptosis, with 68.8% tumor inhibition rates at the highest dosage. HPV16 E6/E7 knockdown in the treatment group was confirmed by RT-PCR to reach 80% and immunohistochemistry indicated elevated levels of p53, p21, and HLA-A proteins. The study establishes that this combination therapy with ENB101-LNP plus cisplatin has a high potential in cervical cancer management, thus offering a therapeutic possibility for targeting HPV16 oncoproteins and reducing contact with side effects associated with existing chemotherapy agents.^110^

 Effective delivery remains the major hurdle in translating cancer gene therapy to the clinic. Ideal gene delivery systems must shield the therapeutic agents from degradation, reduce immunogenicity, and facilitate targeted delivery to achieve therapeutic efficacy without toxicity to other tissues in the body. Studies have shown that PEGylated liposomes have demonstrated enhanced tumor infiltration of small molecular weight drugs that represent a suitable platform for the delivery of nucleic acids. The polyethylene glycol (PEG) coating helps to conceal genetic material from the immune system, rendering it incapable of being cleared from circulation early.^111,112^ In a research study, polyethyleneimine (PEI), which is a cationic polymer, was complexed with siRNA before encapsulation in PEGylated liposomes. Various methods and material compositions were explored to optimize this process. The encapsulated siRNA was designed to target HPV genes to treat cervical cancer. The liposomes were further operated with AG86, an α6β4 integrin-specific peptide-amphiphile, a biomarker for cervical carcinoma. This targeting approach, combined with polymer complexation, was found to be essential for effective transfection and gene silencing. The study underscores the perspective of PEGylated liposomes for transporting therapeutic siRNA specifically to HPV-infected cells, offering a promising strategy for cervical cancer treatment and addressing key issues in gene therapy delivery.^113^

**Figure 4:**
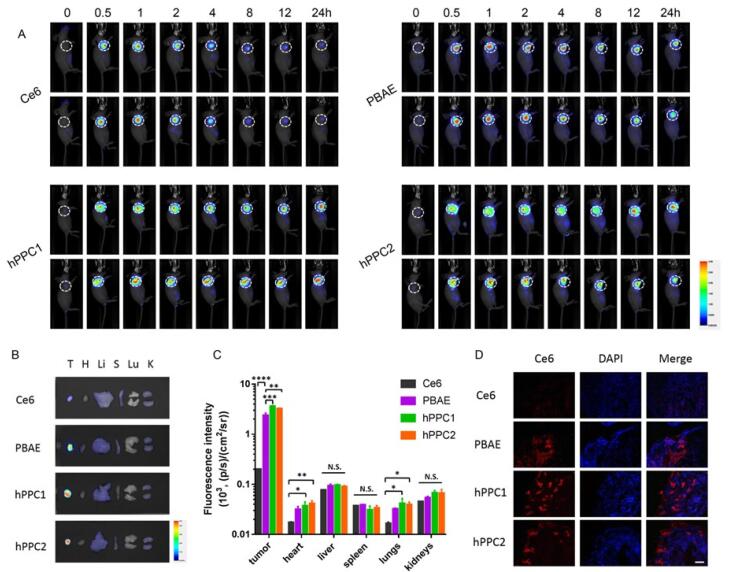
Biodistribution of hPPCs/ polyplex NPs assessed preclinically. (A) *In vivo* fluorescent visualization of SiHa tumor-xenografted mice post-peritumoral injection of free Ce6 and various Ce6-loaded polyplex NPs at multiple time points. (B) Fluorescent photomicrographs of main organs, including tumor, heart, liver, spleen, kidney, and lungs. (C) Quantified fluorescence intensities in organs at 24 hours post-injection. (D) Representative frozen tissue sections of tumors at 24 hours post-treatment, with tumor regions indicated.^118^

 Genome editing is a key tool for research and treating genetic diseases, with CRISPR/Cas9 shown to be highly effective in editing genes both *in vivo and in vitro*. For cervical cancer therapy, targeting HPV driver genes with CRISPR/Cas9 offers a promising approach.^114,115^ Research studies have shown, gene editing tools can restore proteins like p53 and pRb by correcting E6/E7 oncogenes. Delivery of Cas9 as DNA, mRNA, or protein, with mRNA offering more efficient transfection and reduced off-target effects. However, there remains a need for improved non-viral vectors for safe *in vivo* applications.^116^ Therefore, the development of CRISPR/Cas9-mediated nanotherapies for genome editing for cervical carcinoma, targeting HPV’s E6 and E7 oncogenes, was developed by a group of researchers. A pH-responsive hybrid nonviral nano-vector using low molecular weight polyethyleneimine and acetylated cyclic oligosaccharide (ACD), resulting in ACD NP, was designed. These NPs efficiently delivered guide RNAs (gRNAs) and Cas9 mRNA targeting the E6 or E7 oncogenes, creating two therapies: E6/ACD NP and E7/ACD NP. In HeLa cells derived from cervical carcinoma, the ACD NP exhibited high transfection efficiency with low cytotoxicity and successfully edited the target oncogenes with low off-target activity. In mouse models with HeLa xenografts, these nano-therapies showed significant antitumor activity by effectively editing the oncogenes. Additionally, by remodeling tumor-mediated immunosuppression, they ameliorated CD8 + T-cell survival and produced synergistic anticancer activity in combination with adoptive T-cell therapy. The research concludes that these pH-responsive genome editing nano-therapies are promising candidates for treating HPV-associated cervical cancer.^117^ A similar study formulated a non-viral gene carrier using hyperbranched copolymer (hPPC) based polyplex NPs for the administration of the CRISPR/Cas9 system, precisely targeting the oncogenic HPV E7 in HPV-positive cervical carcinoma cells, as shown in Figure 4. The highly branched hPPC1 demonstrated strong plasmid condensation capabilities and proved effective for delivering the CRISPR/Cas9-mediated genome editing platform. By cleaving the HPV E7 oncogene and reducing E7 protein levels, the hPPC-therapeutic plasmid NP system exhibited remarkable anti-tumor effects both in vivo and in vitro, offering a promising new approach for gene-based treatments of HPV-related cervical cancer.^118^

 HPV-16/18 is analogous to numerous cancers of the cervix, oropharynx, anus, penis, and other regions. There are currently preventive vaccines available, but therapeutic vaccines continue to be urgently needed because the high-risk HPVs persistently infect and generate precancerous lesions.^119^ A recent investigation introduced Mhtv-o3E2, an mRNA-based therapeutic vaccine developed with LNPs directed at the E2, E6, and E7 oncoproteins of HPV16 and HPV18. The vaccine produced strong antigen-specific immune responses, particularly activating CD8 + T cells, which resulted in significant extended survival and tumor regression in a mouse model. The immune response was dose-dependent, with higher doses yielding better outcomes. Additionally, the vaccine provided long-lasting T-cell immunity, offering protection against tumor relapse over four months following vaccination. mHTV-03E2 also demonstrated synergy with checkpoint inhibitor therapy, enhancing tumor growth suppression and further extending survival, indicating its potential as part of combination therapy. Overall, the study suggests that mHTV-03E2 is a potential therapeutic agent for managing HPV16 and HPV18-related malignancies, combining the strengths of mRNA technology and LNP formulation to stimulate robust and durable immune responses against existing HPV-associated cancers.^120^

 Another similar investigation presents a therapeutic mRNA-based vaccine, mHTV-02, fabricated with LNP focused on the E6 and E7 proteins of HPV16 and HPV18. Unlike preventive vaccines, mHTV-02 aims to address existing lesions and high-risk HPV infections. In preclinical mouse models, the vaccine elicited a strong antigen-specific immune response, notably activating CD8 + T cells, which resulted in extended survival and significant tumor regression. Additionally, mHTV-02 effectively established long-lasting memory T-cell immunity. The study evaluated various administration routes and found that intramuscular or intratumoral injections of mHTV-02 were highly effective, resulting in substantial therapeutic benefits with 40% complete regression of established tumors. Conversely, intravenous delivery of the vaccine showed minimal impact on tumor reduction and survival improvement. The study highlights the critical role of administration routes in maximizing vaccine efficacy, suggesting that intramuscular or intertumoral delivery methods should be prioritized for optimal therapeutic outcomes.^121^ An mRNA-based vaccination was created by Kun and the team to investigate therapeutic strategies directed against the HPV16 E6 and E7 oncoproteins within high-grade squamous intraepithelial lesions (HSILs), a stage that comes before cervical cancer. The vaccine demonstrated functionality in both *in vivo* and *in vitro* analysis, eliciting a robust antigen-specific immune response. In mice with HPV16 + lesions, vaccination resulted in tumor retardation, prolonged survival up to 100%, and the formation of immunological memory. After the effectiveness of mRNA vaccines for SARS-CoV-2, this mRNA-based therapeutic vaccine shows potential as a non-invasive treatment option for HPV16 + HSILs. It offers a promising alternative to current treatment standards by targeting existing infections and precancerous lesions directly.^122^

 In a recent study, 418 Mexican women between the ages of 25 and 65 who were referred for coloscopy because of abnormal cytology results were asked to evaluate the efficacy of self-sampling in conjugation with a 7-type HPV mRNA E6/E7 test for enhancing cervical carcinoma screening. Participants used self-sampling to collect samples, which were then analyzed with both the 7-type HPV mRNA E6/E7 test and the 14-type HPV DNA test. The study compared the tests in terms of positive predictive value, specificity, sensitivity, and the colposcopy rate needed to determine CIN3 + lesions. The 7-type HPV mRNA E6/E7 test demonstrated non-inferior sensitivity and superior specificity (45.8%, *p*< 0.001) and PPV compared to the 14-type DNA test in the detection of CIN3 + lesions.As a result, colonoscopy rates for CIN3 + cases decreased from 16.6 to 7.6 (*P* < 0.001). Self-testing was well-received by patients, who reported minimal discomfort, receptiveness, and acceptance of self-administering the procedure at home. The integration of self-collected samples with HPV mRNA testing constitutes a novel strategy for improving the sensitivity and specificity of cervical cancer screening with diagnostic precision, potentially reducing morbidity and mortality, particularly in underserved populations.^123^

###  Challenges and limitations of siRNA and mRNA-based strategies 

 The above-discussed studies elaborated on the effectiveness of siRNA and mRNA-based treatment strategies for cervical cancer treatment; however, potential technologies often come with significant treatment challenges in preclinical and clinical studies.

 Firstly, siRNA-based strategies are effective enough to inhibit cancer progression, but the major hurdle is their effective delivery at the site of action, penetration to the vaginal mucosa, and the tumor microenvironment.^124^ Secondly, siRNA is prone to degradation and requires protection either by encapsulation or by chemical modification. Nanocarrier encapsulation may also help to enhance its cellular uptake, which is obstructed by the negative charge of siRNA.^125^ Some studies also highlighted the silencing of non-targeted genes that leads to siRNA-induced toxicity. It is a critical challenge to target siRNA towards oncogenes without affecting the normal genes.^126^ Activation of innate immune responses leads to immunogenicity and other toxicities.^127^ The silencing done by siRNA is temporary, which requires recurrent administration to sustain the effectiveness of treatment. The clinical transitions have started; however, regulatory restrictions and the cost of the trial are the major challenges.^125^

 On the other hand, mRNA-based approaches have been used as preventive strategies from diseases. Like siRNA, mRNA is also susceptible to degradation and unstable in the biological environment. So, the major challenges are its effective delivery to the site of action and complicated anatomical barriers. Additionally, overstimulation and maintenance of immunogenicity to manage its safety are also critical parameters.^128^ Industrial scale-up is technically demanding and incurs huge costs. Storage condition also limits its approachability to low-resource settings.^129^ Moreover, no proper clinical evidence is available to prove the superiority of mRNA vaccines over existing HPV vaccines.^128^

## Patents on siRNA therapy for cervical cancer

 Current research on siRNA-based treatment of cervical cancer has culminated in various new patents to eliminate the survivin gene, which is critical for tumor cells to survive and grow. These patents consider the application of siRNA sequences to inhibit the survivin gene to exert both proliferative and apoptotic effects. The research also aims to improve the delivery methods of siRNA and the integration of gene silencing methods for enhanced therapeutic effects. This emerging field of gene therapy holds a lot of potential for the development of better treatments for cervical cancer. Table 5discusses patents related to siRNA therapy for the management of cervical cancer. For instance, patent no.CN105002183A demonstrated that there is a broad-spectrum siRNA sequence for the survivin gene that causes tumor suppression.^130^ In the same way, method CN104531707A revealed the usage of siRNA that targets survivin protein and has medical applications in biomedicine.^131^ Patent CN105176999A presents a specific siRNA sequence made of positive and antisense strands that have been proven effective in inhibiting cervical cancer cells *in vitro.*^132^ CN106046118A is devoted to designing a novel siRNA delivery system derived from amphiphilic molecules, improving both therapeutic efficacy and gene-silencing performance.^133^ Patent CN104531709A discusses the dual-interference siRNA composition by combining VEGF-C-siRNA and survivin-siRNA for the management of cancers.^134^ Lastly, CN103695420A describes a double-chain siRNA molecule to reduce survivin gene expression, which led to apoptosis of tumor cells and decreased survival.^135^ These patents offer notable improvements in cervical cancer gene treatment through siRNA technology to suppress tumor growth.

**Table 5:** Patents related to siRNA therapy for cervical cancer

**S. No.**	**Aim**	**Patent No.**	**Inventors**	**Country**	**Description**	**References**
1.	Inhibition of surviving gene upregulation by siRNA molecule	CN105002183A	Xie Jing, Sun Yating, Li Jianguang, Teng Lesheng, Meng Qingfan, Lu Jiahui, Liu Yanquan, Yutong, Zhang Yang	China	The invention describes the use of a broad-spectrum siRNA sequence which has both inhibitory and anti-tumour effects on cervical cancer cells. The double-stranded siRNA sequence on entering inside the body, inhibits the protein, genes get silenced and effective elimination of expressed target gene to get the therapeutic outcome.	^130^
2.	Inhibition of survivin gene by siRNA sequence	CN104531707A	Teng Lesheng, Xie Jing, Li Jianguang, Li Yuhuan, Lu Jiahui, Liu Da, Li Yujing, Zhou Yulin, Guo Zhihua	China	This invention describes the method of using a broad-spectrum siRNA sequence which has an inhibiting effect on cervical cancer cells. The double-stranded siRNA sequence on entering the body, inhibits the protein, suppresses the genes and also offers the simultaneous medicinal application of siRNA molecules, falling under the technical domain of biomedicine.	^131^
3.	Transferosome loaded with plasmid and Double-strand siRNA to inhibit survivin	CN105176999A	Teng Lesheng, Hao Fei, Li Jianguang, Xie Jing, Meng Qingfan, Lu Jiahui, Liu Yan, Liu Yang, Quan Yutong	China	The invention shows the involvement of siRNA sequence in gene suppression. The sequence is made up of two strands i.e. the positive strand 5'-GGCCCUUGUCUAAGUGCAA-3' and the anti-sense strand 5'-UUGCACUUAGACAAGGGCC-3'. These sequences inhibit the growth of cervical carcinoma cells *in vitro.*	^132^
4.	Survivin gene silencing by siRNA transfer system	CN106046118A	Wang Yuji, Cui Chunying, Xianying	China	This invention is about the creation of a siRNA transfer system for use in the production of therapeutic pharmaceuticals as anticancer genes. As per the methodological study, the amphiphilic molecule S-Beta-carboline-3-acyl-RGDV has an anticancer action called CRV. Instead of phospholipid and cholesterol, an engineered target is incorporated as the film material to construct the liposome’s exterior. An antitumor gene therapeutic drug is used to create a novel siRNA transfer system which stops the development of tumor cells and inhibits the expression of surviving mRNA.	^133^
5.	Inhibition of tumorigenesis and metastasis by siRNA dual-silencing complex	CN104531709A	Ge Yinlin, Liu Yongchao, Xue Meilan, Zhang Jinyu, Zheng Zheng	China	The invention describes an application of siRNA dual silencing complex for blocking tumorigenesis and spread. A combination of an oligonucleotide survivin-siRNA molecule and oligonucleotide VEGF-C-siRNA molecule is formed to create the siRNA dual-interference composition at a mass ratio of (0-2):(2-0). In this experiment, a chemically generated and modified oligonucleotide molecule was used to transfect a transplanted tumor and suppress tumorigenesis and its spread. The two siRNA interferences are used in antioncogene medication and cervical cancer.	^134^
6.	Double-chain siRNA molecule to target cervical cancer	CN103695420A	Wang Tian has the aftermath, Tang Suoqin, Zhang Xiaomin, Zhang Yan, Qu Kuiyao	China	The invention demonstrates a double-chain siRNA molecule which is made up of two nucleotide sequences: the antisense strand is 5'-UUCUUGAAUGUAGAUGC-3' and the sense strand is 5'-GCAUCUCUACAUUCAAGAA-3'. This double-chain siRNA can suppress surviving gene regulation beyond transcription causing tumor cells to undergo apoptosis, inhibiting mitosis and decreasing the survivability of cervical cancer cells.	^135^

 These patents highlight the promising potential of siRNA-based therapies for cervical cancer. However, the journey from patent to clinical application involves substantial challenges. While these patents provide a foundation for innovation, the practical issues and challenges related to commercialization and regulatory processes need careful consideration. One key challenge is the feasibility of translating these patents into clinical practice. Despite promising *in vitro* and preclinical results, siRNA therapies face barriers to clinical and market acceptance.^136^

 From a commercialization perspective, the intellectual property landscape for siRNA therapies is complex, which can make it difficult to develop and market these treatments. Many siRNA technologies are covered by overlapping patents, which can make licensing agreements difficult and delay the development of new products. For example, the FDA-approved siRNA drugs patisiran, givosiran, and lumasiran are each protected by numerous patents—21, 12, and 10, respectively. This shows how complex the patent landscape is for siRNA therapies. This fragmented patent system creates challenges for innovation.^137^ Disputes over patent rights and lengthy negotiations for licensing can delay product development and market entry. Another challenge is scalability. Manufacturing siRNA therapies at a commercial level requires advanced technologies and large financial investments to make the production process cost-effective and reliable.^138^

 Regulatory approval is another major obstacle in the clinical translation of siRNA therapies. Regulatory agencies such as the FDA and the European Medicines Agency (EMA) have strict requirements for approving novel therapeutics. These include demonstrating strong preclinical data, well-designed clinical trials, and maintaining consistent manufacturing standards. For the FDA, submitting an Investigational New Drug application is an essential first step, requiring detailed data on safety, pharmacokinetics, and pharmacodynamics.^139-141^ Similarly, the EMA offers guidance to developers in aligning their studies with regulatory expectations. A specific challenge for siRNA therapies is minimizing off-target effects, where unintended genes could be silenced, leading to harmful side effects.^142,143^ Regulators also require long-term safety data, particularly for therapies using novel delivery systems, which can extend development timelines and raise costs.^144^

 These extended timelines and regulatory complexities increase the financial risks of developing siRNA therapies and bringing these to the market. Companies must carefully plan investments and resources to manage these risks. To address these challenges, collaboration between policymakers and industry leaders is crucial. Streamlining regulatory pathways and creating clear guidelines can help reduce delays and make siRNA therapies more accessible.^145,146^

## Clinical trials: siRNA and mRNA-based diagnostic and therapy modalities

 Clinical trials remain fundamental blocks in moving innovative scientific research findings into viable therapeutic options for human diseases. Considering HPV-mediated cervical cancer, two of the most promising target genes, siRNAs and mRNA-based therapies, are under investigation for their capability to prevent and cure cervical cancer at the molecular level. Numerous clinical trials are now in progress to determine the safety, efficacy, and long-term advantage of using these revolutionary RNA-based therapies for HPV-induced cervical cancer. They represent significant milestones in establishing the potential of siRNA and mRNA therapies as components of future comprehensive cancer management strategies. Contrasting results of these strategies on viral clearance, tumor volume reduction, and survival rates hold the potential to indicate the potential of RNA-based treatments applied in clinical practices. Several clinical trials that investigate the use of siRNA and mRNA platforms, highlighting their designs, primary endpoints, and preliminary results, have been discussed in this section.^147-150^

 Clinical trial based on comparison of HPV E6/E7 mRNA and HPV DNA screening stratification for cervical carcinoma, bearing ID NCT02116920 by Tata Memorial Hospital, focused on developing and standardizing a methodology for detecting mRNA E6/E7 from HPV genotypes 16, 18, 31, 33, and 45 in cervical samples using real-time PCR. The purpose of the study was to compare this mRNA assay’s performance with the established HPV DNA test (HC2) as a secondary screening tool, using colposcopy and biopsy as the reference standard. Women aged 30 to 65, who were non-pregnant and had intact uteri, and who tested positive in primary cervical screening via visual inspection with acetic acid, were included, while those who were pregnant and had undergone hysterectomy in the past or had already been screened with cervical cancer were excluded. A key objective was to assess the proportion of individuals incorrectly identified as positive in the primary screening by comparing the HPV DNA test›s high specificity with the E6/E7 mRNA assay›s results. The sensitivity and specificity of both tests will be evaluated against histopathology (CINs), the gold standard, to determine their efficacy over a fixed time frame. There might be challenges in coordinating multiple teams (screeners, social workers, lab personnel, clinicians) across settings and ensuring standardized interpretation and minimizing inter-observer variability in colposcopy/biopsy readings. The overall aim was to reduce unnecessary treatments, improve patient outcomes, and enhance the affordability of cervical cancer screening by optimizing the triage of women who test positive in primary screenings.^147^

 The clinical trial based on the identification of oncogenic HPV E6/E7 protein in tampon-based self-sampling (ID: NCT00526370) by the University of Aarhus explored the feasibility of using tampon self-tests to detect HPV E6/E7 mRNA as an alternative to traditional cervical smears. The study hypothesised that the self-collected sample’s HPV E6/E7 mRNA would apply to cervical carcinoma screening diagnostics. The main goal of the research was to evaluate the detection abilities of the HPV E6/E7 mRNA test through the information obtained from the tampon self-tests. The sensitivity and specificity of these results were then compared to cytological and histological diagnosis as an initial check on the feasibility and effectiveness of this self-test technique for oncogenic HPV, which could prove to be simpler and less invasive than routine cervical smear. As a cross-sectional study, no intervention was given, and no management plan was implemented, so it centred solely on the assessment made when diagnosing the disease. A total of 100 women were recruited: Half of the sample comprised women aged 50 or less who participated in the national cervical cancer screening program with a GP, and the other 50 women who were first seen at the outpatient gynaecological department because of cervical lesions.148 The inclusion criteria comprise women with cervical dysplasia and recommended for conization, while the women not performed a self-tampon test before conization. The concern regarding this trial is the variation in the quality of tampon-collected samples, and the users might not place them correctly. The small population size may limit the statistical power and confidence in identifying significant differences.

 A multicentre randomized trial (NCT01837693), integrated into Italian cervical cancer screening programs, evaluates the effectiveness of mRNA and p16 as primary screening tests or triage methods following a positive HPV DNA test. The study compares the detection rates of CIN2 + over five years in women with + ve HPV DNA but -ve mRNA or p16 results, which may cause participant attrition over the years and may lose the statistical power. The primary aim was to assess whether mRNA and p16 can reduce overdiagnosis compared to direct colposcopy referrals, improving the specificity of screening while maintaining sensitivity for high-grade lesions. It is crucial to determine a safe risk threshold before excluding colposcopy. Women with positive HPV DNA were tested for cytology, mRNA, and p16; those with negative cytology were randomized into two groups for further evaluation. Secondary objectives included assessing the feasibility of mRNA as a primary screening test, validating sampling techniques, and standardizing quality controls for these new methods. The study involved women aged 25–59 and aimed to refine cervical cancer screening protocols by minimizing unnecessary colposcopies and overdiagnosis.^149^ The clinical trial started in March 2024 entitled «A Study in Subjects with HPV 16 or 18 related CIN 2 or 3» (ID: NCT06273553) by RinuaGene Biotechnology Co., Ltd. aims to investigate the safety profile, acceptability, immunogenicity, and efficacy of the mRNA therapeutic vaccine RG002 in women aged 18-55 with HPV16/18-associated CIN2 or CIN3. In this trial, there are two parts of the informed consent form. Part A should be signed by women of 18-45 years of age; while signing Part B, women should be aged 18-55 years. In addition, women should be diagnosed with CIN Grade 2 or 3, as verified by central pathological reviewers, around 12 weeks before the initial study immunization was administered. This inclusion criterion could be a challenge in recruiting subjects for the trial. The exclusion criteria were the diagnosis of cervical adenocarcinoma in situ, or atypical endometrial or glandular cells, or evidence of invasive cervical carcinoma on cervical biopsy, around 12 weeks before first vaccine administration. This interventional study uses a single-group assignment to administer the RG002 injection over two phases (Parts A and B), monitoring outcomes for potential cervical cancer treatment. The primary outcomes include assessing safety and tolerability through the incidence of adverse events over 9 weeks, along with determining the suggested Phase 2 dose and maximum tolerable dose from weeks 9 to 36. The study also evaluates the primary efficacy of the vaccine, measured by histopathological regression of lesions to CIN1 or normal. Secondary outcomes focus on HPV16/18 clearance, immune responses (cytokine expression, cellular immune activity, and anti-HPV antibodies), and optional biomarker evaluation and immune cell infiltration in lesions.^150^

## Future Prospects

 In the transforming future of cervical cancer, siRNA and mRNA therapies offer the potential for precise, effective targeting and are a less invasive option for treating HPV-induced cervical cancer. HPV-induced cervical cancer can be treated through various treatment strategies like surgeries, chemotherapy, siRNA, and mRNA therapies. Some suggestions can be considered in treating cervical cancer in a more prominent way, like continuing to invest ‎more amount of money in the development of siRNA and mRNA-based therapies, as these approaches specifically target the HPV genes and are less invasive than current treatment. The condition of cervical cancer is more common in low- and middle-income regions where women are not aware of this disease. This disease remains largely unknown to these women, and even those with a basic awareness fail to recognize its significance. The etiology of cervical cancer about HPV is not well understood by the general populace. The prevention and treatment of cervical cancer remains a critical health matter because low-income women deal with substantial challenges in accessing care. These women find it challenging to obtain necessary medical care due to expensive vaccine and treatment costs that exist alongside a risky, unhygienic environment. Public health must intensify its efforts by creating expanded educational programs about the HPV vaccination benefits, which must include equivalent information about its vital nature for both male and female children. The fight against HPV demands intensified efforts toward both low- and middle-income areas since these regions experience greater HPV prevalence rates with insufficient vaccine and educational access. The reduction of vaccine prices along with treatment expenses should remain the top priority. Partnerships between government agencies and pharmaceutical firms, and government subsidies, could together decrease the cost of accessible healthcare services. Vaccine strategies need to extend beyond women to male populations because numerous individuals still believe cervical cancer exclusively affects women. Public awareness must increase about HPV-related cancers because they affect men and women. The use of early detection techniques together with mRNA-based therapeutic approaches demonstrates great potential to decrease cancer progression rates. Current research must evaluate nanoparticle technologies for medicines to improve therapy results and decrease negative consequences during treatment. The fear of cervical cancer severity often leads patients to avoid necessary medical treatment. Selecting siRNA and mRNA therapies should take precedence since they cause less pain and produce fewer side effects. Such treatment methods deliver improved recovery times in addition to better comfort levels, thus strengthening patient trust in their healthcare procedures. Patient trust in their safety, combined with their health status, plays a vital role in achieving better medical treatment results. The therapies provide patients with both accelerated recoveries and better therapeutic experiences, which enhances their confidence in treatments. Patient safety and health must improve for better health results to become achievable. Screening and vaccination efforts should receive heightened attention because 85 per cent of women remain unaware of cervical cancer, even though its initial stages do not display noticeable symptoms. Women from lower and middle-income families should prioritize cervical cancer screening because these communities typically face restricted health service options. The reduction of this disease burden depends on advanced studies about HPV-induced cervical cancer epidemiology and strengthened public health initiatives to lower its mortality rate.

## Conclusion

 HPV types 16 and 18 are responsible for around 75% of cervical carcinoma cases. The continuous HPV infection leads to cancer and causes inactivation of tumour-suppressing proteins, which leads to uncontrolled cell growth. The HPV-induced cervical cancer is challenging to manage in resource-constrained settings, where vaccination and screening strategies are the only way of reducing this cancer. The recent advancements emphasize the role of siRNA and mRNA therapies, which target HPV oncoproteins to suppress cervical cancer cell growth. Many experimental approaches, such as LNPs and CRISPR/Cas9, show effective results in silencing HPV genes and activating immune responses. Current treatment, like surgery, radiotherapy, and chemotherapy, shows effective responses in cervical cancer but sometimes shows side effects. siRNA therapy silences the HPV E6 and E7 oncogenes, which are responsible for disrupting cell cycle regulation, leading to cancer growth. mRNA-based therapeutic vaccines stimulate the immune system by introducing viral antigens, which trigger a targeted immune response to fight against HPV-infected cells. These therapies, combined with advanced delivery systems like PEGylated and NPs, show effective results in both preclinical and clinical trials. The integration of these innovative therapeutic strategies with existing treatment modalities holds great promise for improving outcomes in cervical cancer patients and may pave the way for more effective management of HPV-related malignancies in the future.

## Competing Interests

 The authors declare no conflict of interest.

## Data Availability Statement

 Not Applicable.

## Ethical Approval

 Not applicable.
